# Reduced diversity and altered composition of the gut microbiome in individuals with myalgic encephalomyelitis/chronic fatigue syndrome

**DOI:** 10.1186/s40168-016-0171-4

**Published:** 2016-06-23

**Authors:** Ludovic Giloteaux, Julia K. Goodrich, William A. Walters, Susan M. Levine, Ruth E. Ley, Maureen R. Hanson

**Affiliations:** Department of Molecular Biology and Genetics, Cornell University, Ithaca, NY USA; Department of Microbiology, Cornell University, Ithaca, NY USA; Private Practice, New York, NY USA

**Keywords:** Myalgic encephalomyelitis, Chronic fatigue syndrome, Inflammation, Lipopolysaccharides, Microbiome, Microbial translocation, Beta-diversity

## Abstract

**Background:**

Gastrointestinal disturbances are among symptoms commonly reported by individuals diagnosed with myalgic encephalomyelitis/chronic fatigue syndrome (ME/CFS). However, whether ME/CFS is associated with an altered microbiome has remained uncertain. Here, we profiled gut microbial diversity by sequencing 16S ribosomal ribonucleic acid (rRNA) genes from stool as well as inflammatory markers from serum for cases (*n* = 48) and controls (*n* = 39). We also examined a set of inflammatory markers in blood: C-reactive protein (CRP), intestinal fatty acid-binding protein (I-FABP), lipopolysaccharide (LPS), LPS-binding protein (LBP), and soluble CD14 (sCD14).

**Results:**

We observed elevated levels of some blood markers for microbial translocation in ME/CFS patients; levels of LPS, LBP, and sCD14 were elevated in ME/CFS subjects. Levels of LBP correlated with LPS and sCD14 and LPS levels correlated with sCD14. Through deep sequencing of bacterial rRNA markers, we identified differences between the gut microbiomes of healthy individuals and patients with ME/CFS. We observed that bacterial diversity was decreased in the ME/CFS specimens compared to controls, in particular, a reduction in the relative abundance and diversity of members belonging to the Firmicutes phylum. In the patient cohort, we find less diversity as well as increases in specific species often reported to be pro-inflammatory species and reduction in species frequently described as anti-inflammatory. Using a machine learning approach trained on the data obtained from 16S rRNA and inflammatory markers, individuals were classified correctly as ME/CFS with a cross-validation accuracy of 82.93 %.

**Conclusions:**

Our results indicate dysbiosis of the gut microbiota in this disease and further suggest an increased incidence of microbial translocation, which may play a role in inflammatory symptoms in ME/CFS.

**Electronic supplementary material:**

The online version of this article (doi:10.1186/s40168-016-0171-4) contains supplementary material, which is available to authorized users.

## Background

Myalgic encephalomyelitis (ME), also known as chronic fatigue syndrome (CFS), or ME/CFS, is a debilitating illness of unknown etiology with no widely accepted therapy. Primary symptoms reported by patients are fatigue, muscle and/or joint paint, sore throat, headaches, unrefreshing sleep, and post-exertional malaise and have been the basis of the widely used Fukuda diagnostic criteria [1]. Many ME/CFS patients also report gastrointestinal (GI) symptoms, including but not limited to irritable bowel syndrome (IBS) [2–6]. Intestinal discomfort is also indicated in a survey of drug use by individuals with CFS compared to controls, which found significantly more use of antacids, H2 blockers, and proton pump inhibitors in the ME/CFS cohort [7].

The prevalence of bowel symptoms has led to attempts to treat the disease by probiotic oral or rectal supplements. Borody et al. [8] reported improvements in a majority of patients at 4 weeks following bacteriotherapy comprised of rectal infusion of 13 enteric bacteria, though the number with a sustained response was not well documented. In two small studies, marginal improvement in certain symptoms was reported following oral probiotic therapy [9, 10].

Two reports suggest altered gut microbiota in ME/CFS patients. Using culture-based methods, Sheedy et al. [11] described higher levels of D-lactic acid producing *Enterococcus* and *Streptococcus* spp. in ME/CFS patients vs. controls. More recently, Norwegian ME/CFS patients and healthy controls were found to exhibit differences in gut microbiota composition through a 16S rRNA gene sequencing study [12]. It is well documented that gut microbiota can be significant with respect to pathological intestinal conditions such as ulcerative colitis (UC), Crohn’s disease (CD) [13], and systemic diseases such as diabetes [14]. Because of the frequent occurrence of GI disturbances, as well as these prior reports of abnormalities, we investigated the diversity and composition of the gut microbiota of ME/CFS patients in comparison to healthy individuals.

Along with GI symptoms, individuals with ME/CFS appear to have both immune activation and immune dysfunction. Many of the common symptoms reported by ME/CFS patients are characteristic of inflammatory illnesses [15]. Most reports concerning cytokine levels in ME/CFS patients vs. controls are somewhat limited in scope and discordant, but several recent papers with a 51-plex cytokine assay indicate abnormal immune signatures in plasma and cerebrospinal fluid [16, 17].

Abnormal immune activation can be caused by translocation of microbes from an inflamed gut [18]. A prior report indicated increased IgA and IgM to lipopolysaccharide (LPS) in serum of CFS patients [19]. We therefore assayed plasma levels of LPS and LPS-binding protein, as well as the LPS/LBP receptor sCD14 [20]. We also examined the levels of C-reactive protein, an inflammatory marker, and I-FABP as a marker for gastrointestinal tract integrity [21].

Objective molecular markers for diagnosis of ME/CFS are lacking. We examined the levels of plasma markers and microbiota composition in the diseased vs. healthy subjects in order to determine whether the data, taken together, could predict ME/CFS vs. healthy status.

## Results

### Study population characteristics

Subjects with ME/CFS were established patients of a ME/CFS specialist, Susan Levine, M.D. and fit the Fukuda diagnostic criteria [1]. This study began before the criteria for systemic exertion intolerance disease (SEID) were established [22], but most, perhaps all, also fit the description of SEID. Of the 48 patients and 39 control participants who self-reported good health, 34 ME/CFS patients and 7 controls self-reported gastrointestinal disturbances such as constipation, diarrhea, or intestinal discomfort. Many ME/CFS patients are able to identify an acute, often flu-like, illness that immediately preceded the onset of the disease eventually diagnosed as ME/CFS, while others are unaware of an initiating event and consider their onset to be gradual. Among the 48 ME/CFS patients in the study, 19 indicated a gradual and 25 stated a sudden onset. ME/CFS subjects completed the SF-36 form (Additional file 1: Figure S1) and Bell’s Disability scale (Table 1).

**Table 1:** Characteristics of the study population

		Controls (*n* = 39)	ME/CFS (*n* = 49)
Gender	Female	30	38
	Male	9	11
Age	Mean ± SD	45.5 ± 9.9	50.2 ± 12.6
	Median (range)	48 (20–61)	51 (19–71)
BMI	Mean ± SD	27.1 ± 6.1	25.5 ± 4.9
	Median (range)	26 (17–47)	24.5 (16–40)
Bell’s disability scale	10–20	NA	15
	30–40		21
	50–60		9
	>60		4

In comparison to other studies in which patients diagnosed with ME/CFS also filled out the SF-36 form, our study population fell within the same ranges on the eight subscales of the SF-36 (Additional file 1: Figure S1).

### Measurements of levels of microbial translocation markers indicate microbial translocation

**Fig. 1:**
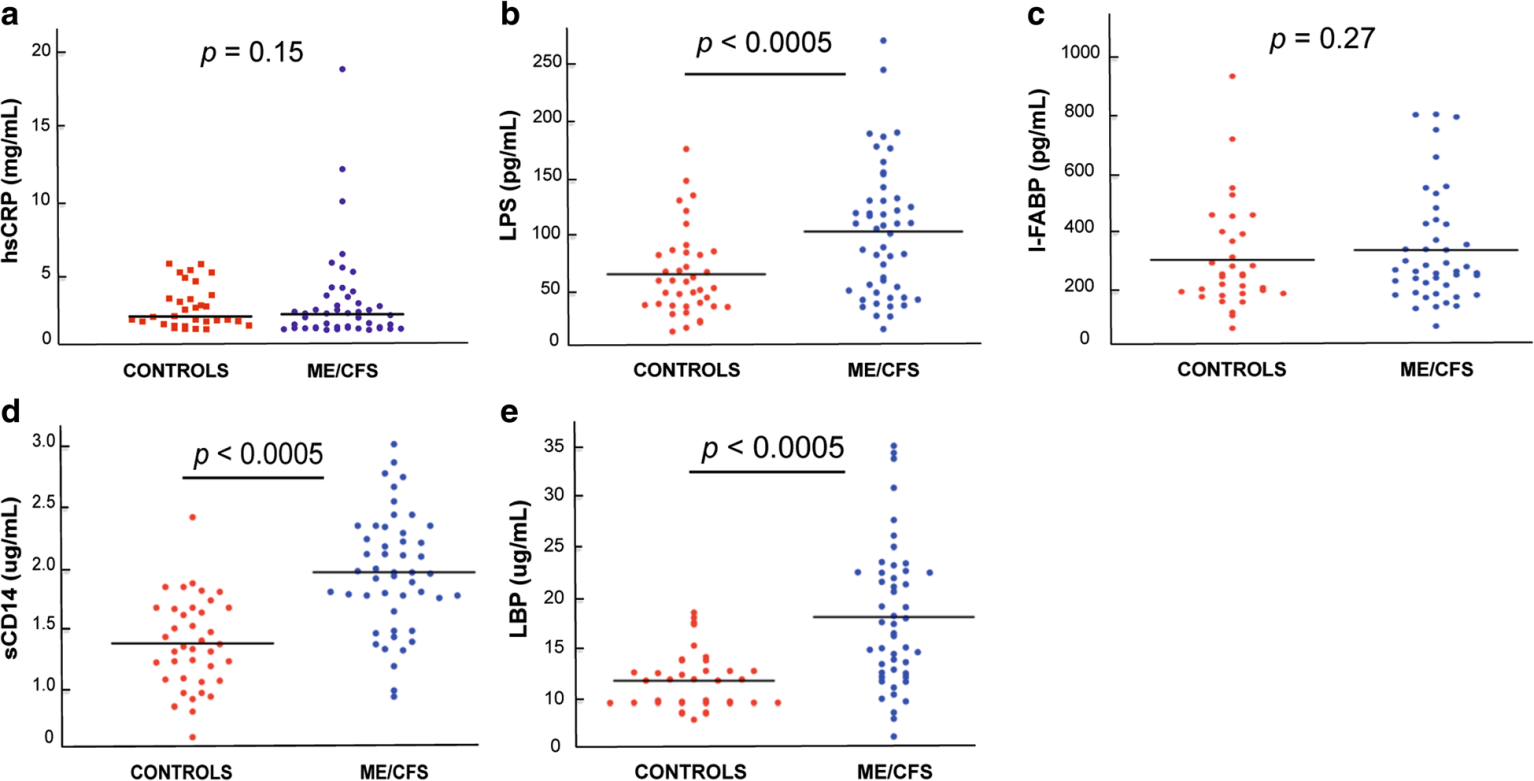
Microbial translocation, gastrointestinal tract damage, and evidence for direct LPS stimulation in vivo in ME/CFS: plasma levels of hsCRP (**a**), LPS (**b**), I-FABP (**c**), sCD14 (**d**), and LBP (**e**) determined in our cohorts of controls and ME/CFS diagnosed individuals. *p* values were calculated by the Wilcoxon-Mann-Whitney *U* test

We quantified plasma levels of hsCRP, lipopolysaccharides (LPS) as a marker of microbial translocation (MT) and plasma intestinal fatty acid binding protein (I-FABP) as a marker for gastrointestinal tract damage in both groups. The distribution of plasma hsCRP, LPS and I-FABP is shown in Fig. 1. Levels of hsCRP were higher in the ME/CFS population in comparison to healthy controls (1.38 and 1.21 mg/L, respectively), but the difference was not statistically significant (*P* = 0.15, Fig. 1a, Table 2).

**Table 2:** Plasma levels of markers of inflammation (hsCRP), microbial translocation (LPS, sCD14, and LBP) and gastrointestinal damage (I-FABP) in ME/CFS and healthy individuals

Analytes	Control	ME/CFS
hsCRP (mg/L)		
*n*	34	46
Median	1.21	1.38
Quartiles	0.38–2.69	0.70–2.71
Range	0.27–5.09	0.23–19.3
LPS (pg/mL)		
*n*	39	49
Median	74.74	119.43
Quartiles	54.34–99.54	66.21–144.41
Range	32.21–187.32	34.32–279.30
I-FABP (pg/mL)		
*n*	33	44
Median	234.40	255.85
Quartiles	159.75–412.90	171.275–450.70
Range	14–1067.9	23.2–909.9
sCD14 (ug/mL)		
*n*	39	49
Median	1.36	1.97
Quartiles	1.09–1.67	1.71–2.32
Range	0.62–2.42	0.95–3.02
LBP (ug/mL)		
*n*	37	49
Median	12.32	17.68
Quartiles	10.22–13.73	13.04–22.56
Range	8.65–18.76	7.06–34.52

ME/CFS patients had significantly higher plasma LPS levels than healthy individuals (median ME/CFS—119.43 pg/mL vs. controls—74.74 pg/mL, *P* < 0.0005, Fig. 1b and Table 2). The median plasma I-FABP level was 341.9 pg/mL in the ME/CFS group and 301 pg/mL in the healthy group. Though the median I-FABP levels in the ME/CFS group was higher than that of the healthy group, the difference was not statistically significant (*P* = 0.27, Fig. 1c, Table 2).

To obtain further information concerning chronic LPS stimulation in vivo, we also measured plasma sCD14 levels and plasma LBP, which is produced by gastrointestinal and hepatic epithelial cells. Thus, increased LPS in the circulation promote hepatic synthesis of LBP, a plasma protein that increases the binding of LPS to CD14. sCD14 and LBP concentrations in both groups are shown in Fig. 1. For the ME/CFS cohort the median plasma sCD14 concentration was 1.97 ug/mL, and the median LBP plasma concentration was 17.68 ug/mL. These values were significantly different from the plasma sCD14 and LBP concentrations of the healthy volunteers (1.36 ug/mL; *P* < 0.0005 and 12.32 ug/mL; *P* < 0.0005, respectively) (Fig. 1d, e, Table 2).

**Fig. 2:**
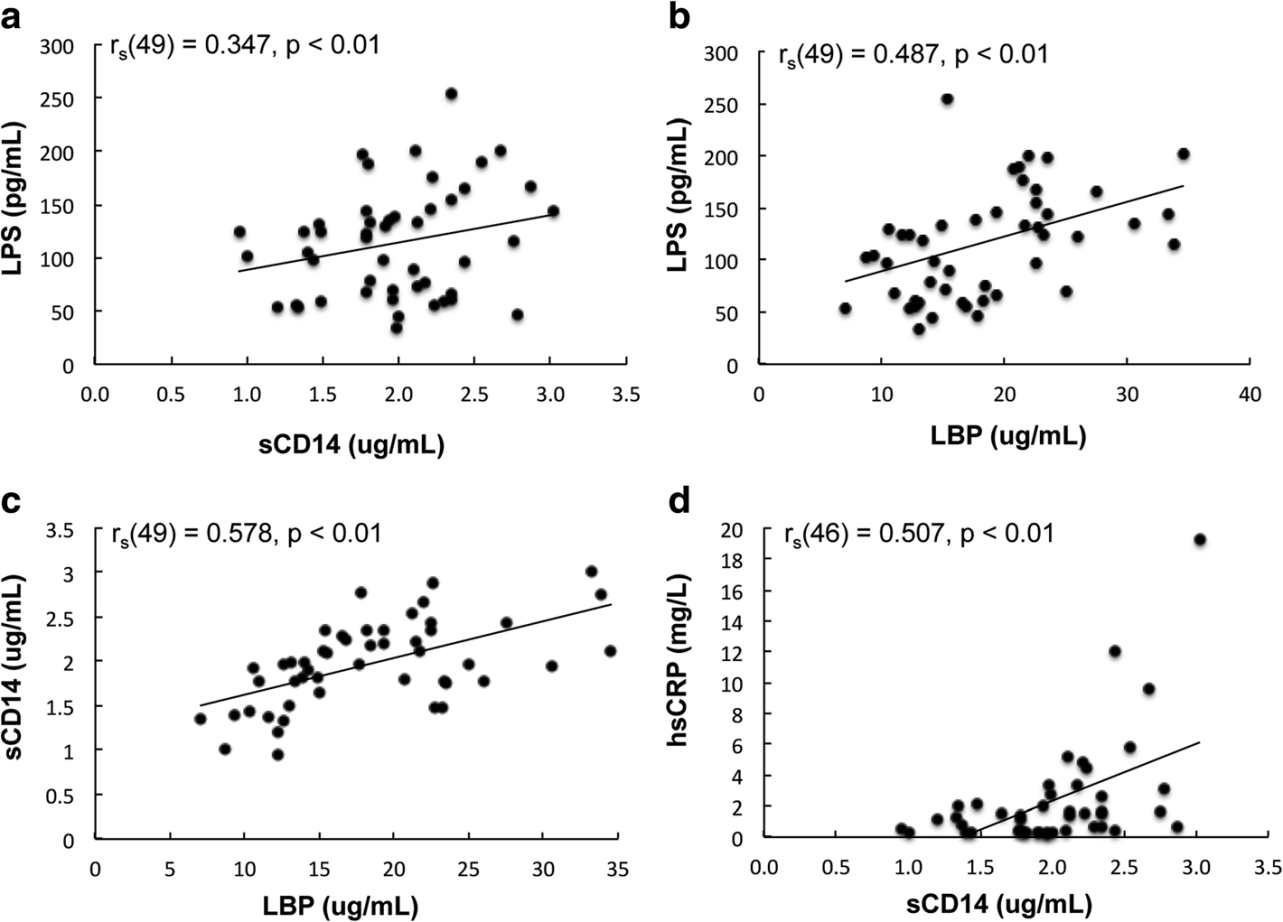
Correlation between plasma levels of LPS and sCD14 (**a**), plasma levels of LPS and LBP (**b**), plasma levels of sCD14 and LBP (**c**), and plasma levels of hsCRP and sCD14 (**d**) in the ME/CFS population. Spearman’s rank test was used to determine correlations

Next, we analyzed the associations among biomarker measurements in the ME/CFS population. As can be seen in Fig. 2a, b plasma LPS levels correlated positively with levels of sCD14 and LBP (*r =* 0.347, *P <* 0.01 and *r* = 0.487, *P <* 0.01, respectively), consistent with stimulation of sCD14 production by LPS in vivo. In addition, we found a strong significant correlation between plasma sCD14 and hsCRP and sCD14 and LBP; high levels of sCD14 were associated with high levels of hsCRP (*r* = 0.507, *P* < 0.01) and LBP (*r* = 0.578, *P* < 0.01) (Fig. 2c, d). We also analyzed whether enterocyte damage (i.e., I-FABP levels) was associated with the proposed microbial translocation markers LPS, sCD14, and LBP. We found no relationship between I-FABP and LPS levels (*r* = −0.125; *P* = 0.278), I-FABP and sCD14 levels (*r* = −0.117; *P* = 0.310), or I-FABP and LBP levels (*r* = −0.08; *P* = 0.488).

### Stool microbiota of ME/CFS patients exhibit reduced diversity and different composition than healthy controls

**Fig. 3:**
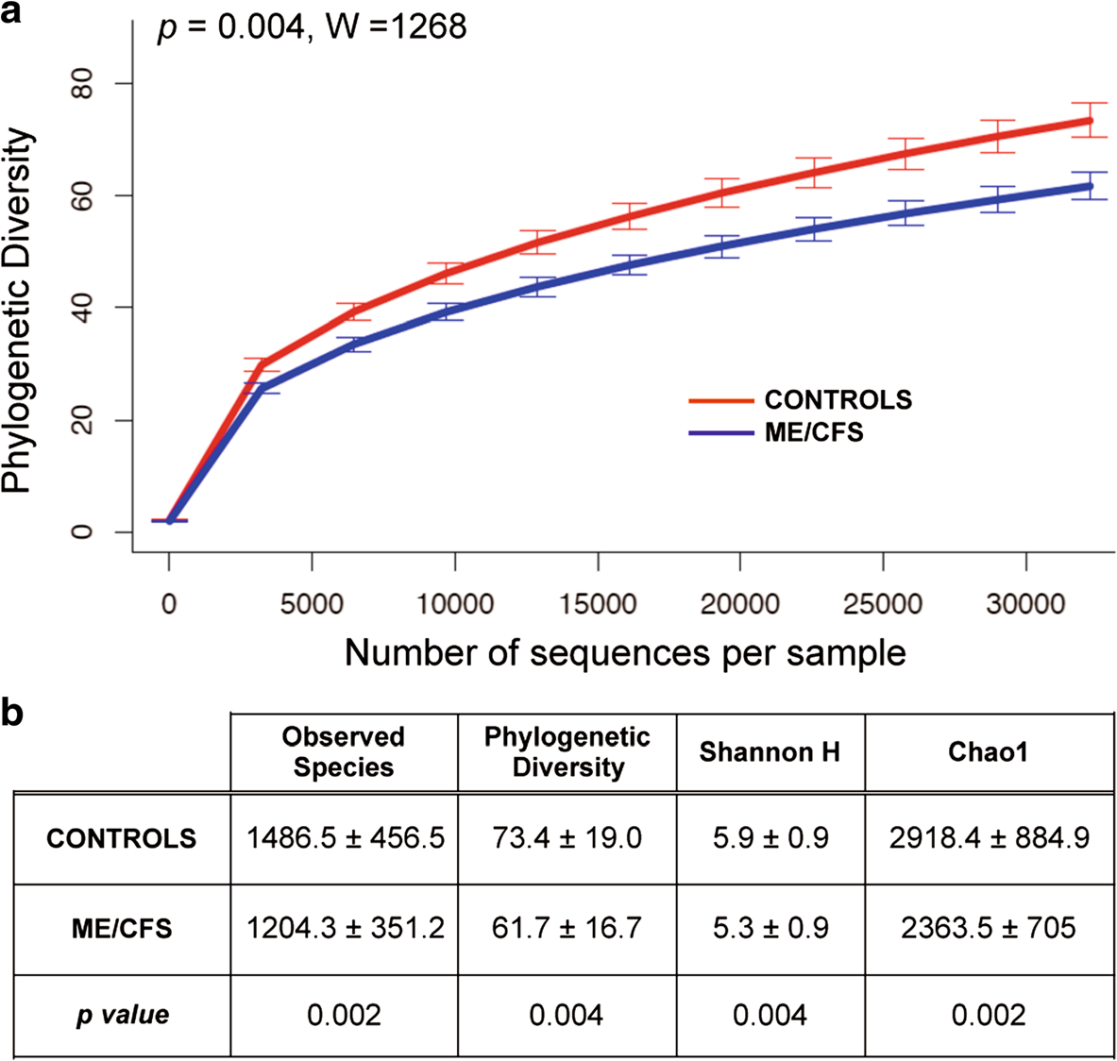
Rarefaction curves and confusion matrix. **a** Rarefaction curves for the microbiota of healthy individuals and ME/CFS patients (each group was rarefied to the number of sequences of the less-sequenced sample, i.e., 32223 sequences). The *p* value was calculated by the Wilcoxon rank-sum test and **b** comparison of alpha diversity indexes in ME/CFS and healthy individuals

The hypervariable V4 region of 16S rRNA genes was sequenced from fecal samples of individuals with ME/CFS (*n* = 48) and healthy individuals (*n* = 39). A total of 8,534,117 high-quality and classifiable reads were generated from all samples, with an average of 98,093 ± 29,231 reads per sample. Binning sequences using a pairwise identity threshold of 97 %, we obtained an average of 1330 ± 423 operational taxonomic units (OTUs) per sample. The sequence-based rarefaction curves based on the Phylogenetic Diversity (PD) metric were nearly asymptotic and a Wilcoxon rank-sum test demonstrated a statistical difference in the diversity of ME/CFS and healthy individuals (*P* = 0.004, W = 1268) (Fig. 3a).

We examined the number of “observed species,” i.e., the number of 97 % ID OTUs observed in 32,223 sequences, the estimators of community evenness (Shannon H), and richness (Chao1 and PD) in the two group of samples. ME/CFS samples had a significant overall lower microbial diversity, with lower evenness (*H* = 5.33 ± 0.93 vs. 5.92 ± 0.93, *P* = 0.004), and lower richness (observed species, 1204 ± 351 vs. 1486 ± 456; Chao1, 2363 ± 704 vs. 2918 ± 885, *P* = 0.002; PD, 61.6 ± 16.7 vs. 73.4 ± 19.04, *P* = 0.004) (Fig. 3b).

To evaluate overall differences in beta-diversity between the microbiomes, we applied Principal Component Analysis (PCoA) to weighted and unweighted UniFrac distance metric matrices generated for the sample set. Within the microbial community cluster, there appears to be no clear difference in beta-diversity between the ME/CFS group and healthy group using both weighted (Additional file 2: Figure S2a) and unweighted (Additional file 2: Figure S2b) UniFrac distance matrices. None of the other parameters tested, i.e., sex, BMI, or clinical data revealed clustering (data not shown). Because beta-diversity clustering as measured by UniFrac shows how dissimilar overall community structure is between samples, the samples may not cluster in a manner that reflects differences detected at the OTU level, or the overall alpha diversity within groups.

**Fig. 4:**
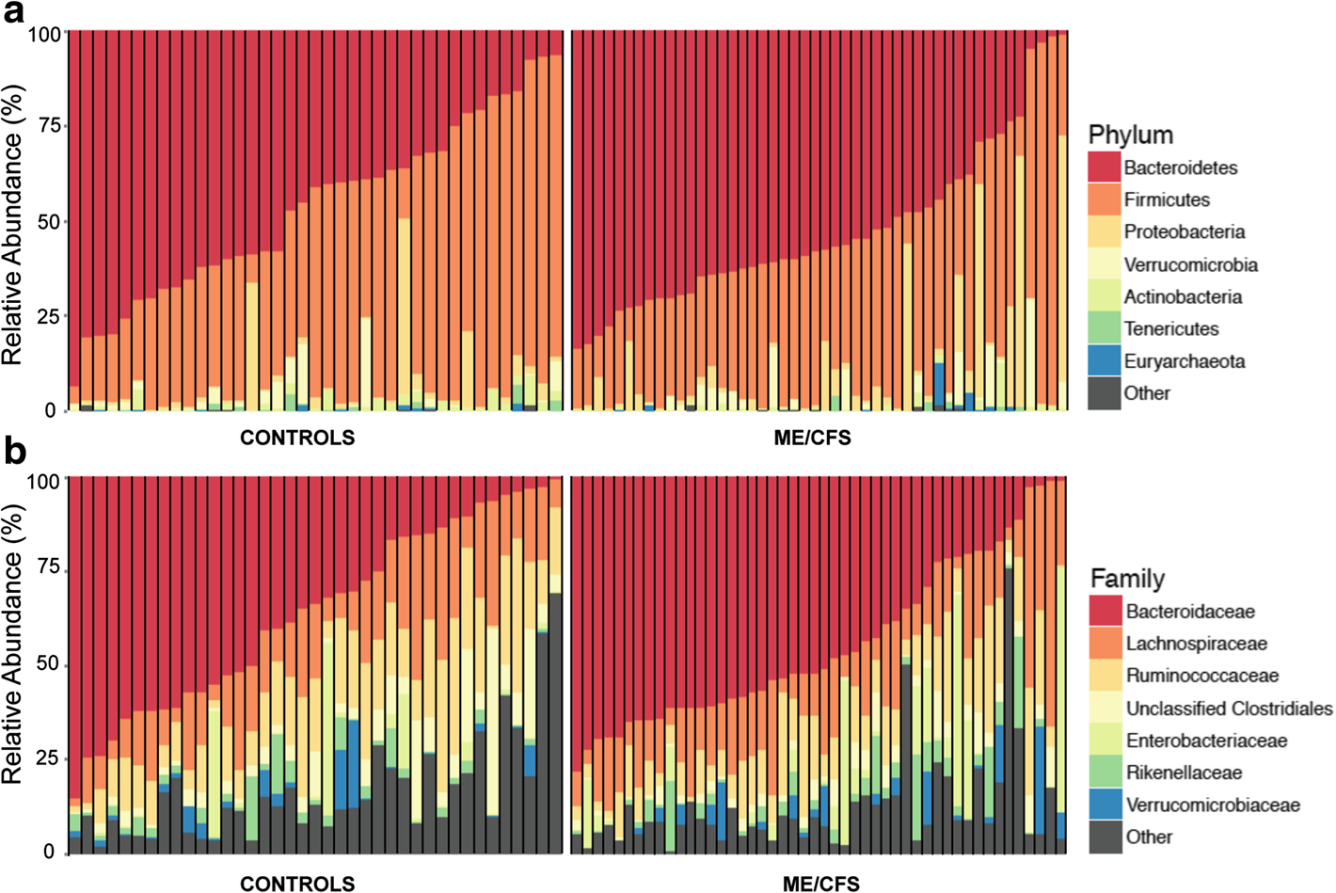
Composition of the gut microbiome of healthy individuals and ME/CFS patients. Relative abundance of phylum-level (**a**) and family-level (**b**) gut microbial taxa

The overall microbial composition for ME/CFS and controls differed at the phylum and family levels (Fig. 4a, b), although none of these differences were statistically significant after multiple test correction. The two largest phyla represented in each dataset of healthy and ME/CFS-afflicted individuals were Firmicutes and Bacteroidetes. In healthy individuals, this corresponded to 46 and 45 % respectively of the rarified 16S rRNA sequences. Also, Proteobacteria made up the next largest represented phylum (3.6 %), with Verrucomicrobia and Actinobacteria in relatively low relative abundance (2.1 and 1.6 %, respectively). At the phylum level, the abundance of the Bacteroidetes was comparable (52 %) in both datasets (Fig. 4a). ME/CFS samples showed lower relative abundance of Firmicutes (35 %) (Fig. 4a) and higher relative abundance of Proteobacteria (8 %), due almost entirely to a twofold increase in the Proteobacteria family Enterobacteriaceae (6 vs. 3 % for ME/CFS and healthy individuals, respectively) (Fig. 4b). Within the Firmicutes, at the family level, Ruminococcaceae were lower in the ME/CFS samples (16 vs. 11 % in ME/CFS and healthy individuals respectively) (Fig. 4b), whereas Lachnospiraceae were similar among both datasets (16 % for both healthy and ME/CFS samples). Some differences were noted between cases and controls in family members of the Bacteroidetes, i.e., Bacteroidaceae (35 vs. 43 %), Rickenellaceae (3 vs. 4 %), and Prevotellaceae (3.2 vs. 0.7 %). Finally, within the Actinobacteria, Bifidobacteriaceae were lower in the ME/CFS samples (1 vs. 0.5 %).

At the OTU level, 40 OTUs were found to be significantly different between groups after multiple testing correction. The majority of them belonged to the Firmicutes phylum, including members of the Ruminococcaceae family such as *Oscillospira* spp. (*q* = 0.016), *Faecalibacterium prausnitzii* (*q* = 0.014), and *Ruminococcus* spp. (*q* = 0.014) and members of the Lachnospiraceae, i.e., *Coprococcus* spp. (*q* = 0.014). Other OTUs included members of the Actinobacteria such as *Eggerthella lenta* (*q* = 0.014) and *Collinsella aerofaciens* (*q* = 0.014).

**Fig. 5:**
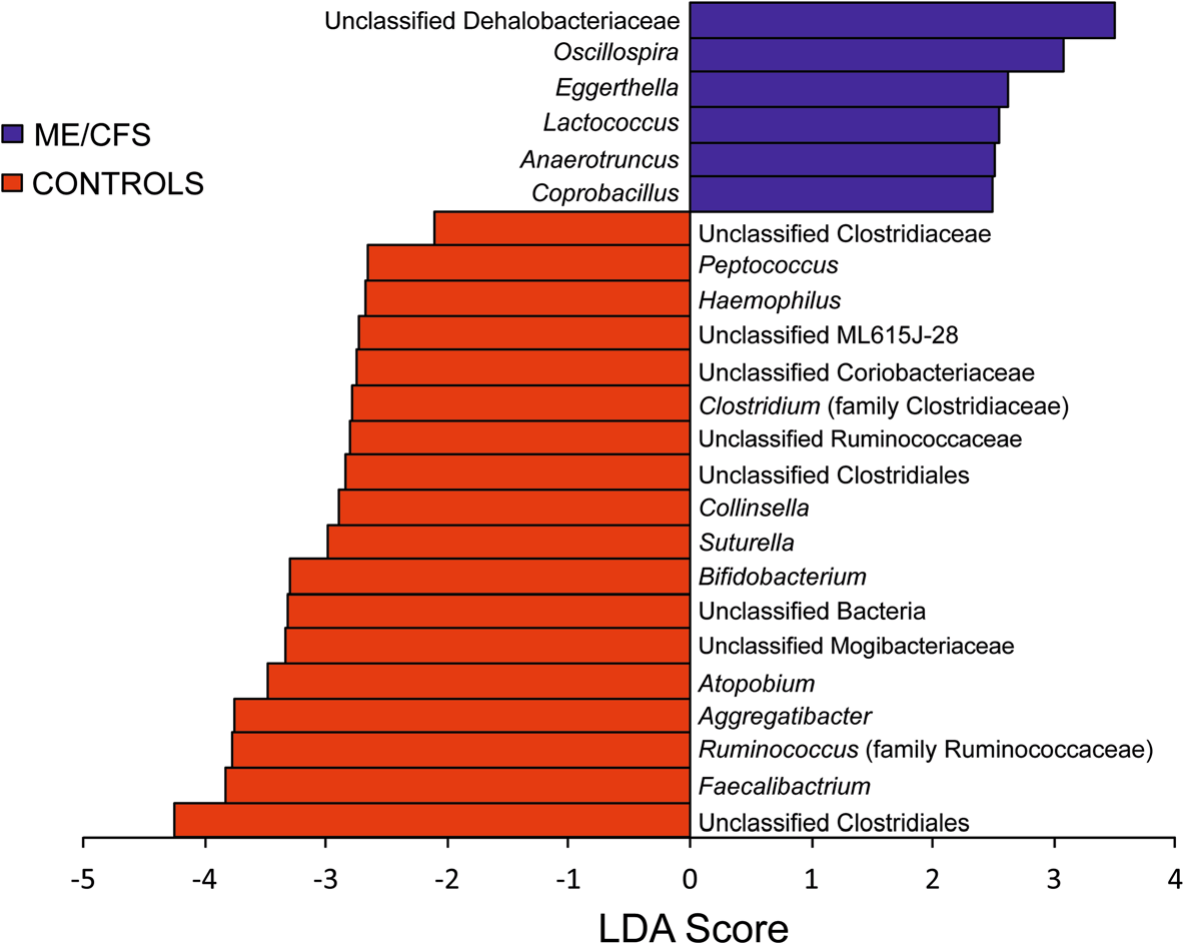
Histogram of the LDA scores computed for genera differentially abundant between ME/CFS and healthy individuals. ME/CFS-enriched genera are indicated with a positive LDA score, and genera enriched in healthy individuals have a negative score. The LDA score indicates the effect size and ranking of each differentially abundant taxon

These significant differences were further confirmed by LEfSe analysis, which uses linear discriminant analysis (LDA) coupled with effect size measurements to identify bacterial taxa whose sequences are differentially abundant between ME/CFS and healthy individuals. In addition to detecting significant features, LEfSe also ranks features by effect size, which put features explaining most of the biological difference at top (Segata et al. 2011). LEfSe identified 24 discriminative features (genus level, LDA score >2) whose relative abundance varied significantly among fecal samples taken from the ME/CFS and healthy groups (Fig. 5). ME/CFS microbiota were enriched with an unclassified member of the Desulfohalobacteriaceae and genera from the Firmicutes phylum, i.e., *Oscillospira*, *Lactococcus, Anaerotruncus* and *Coprobacillus* and *Eggerthella*, a member of the Actinobacteria phylum (*P* < 0.05, Fig. 5). Eighteen genera were enriched in the control group compared to the ME/CFS group (Fig. 5) with members mainly belonging to the Firmicutes phylum. We observed that members of the Ruminococcaeae and Bifidobacteriaceae, i.e., *Faecalibacterium* and *Bifidobacterium*, respectively, were significantly increased in healthy individuals (*P* = 0.03 and 0.04, respectively).

### Classifying subjects into patients vs. controls from inflammatory markers and microbiome data

**Fig 6:**
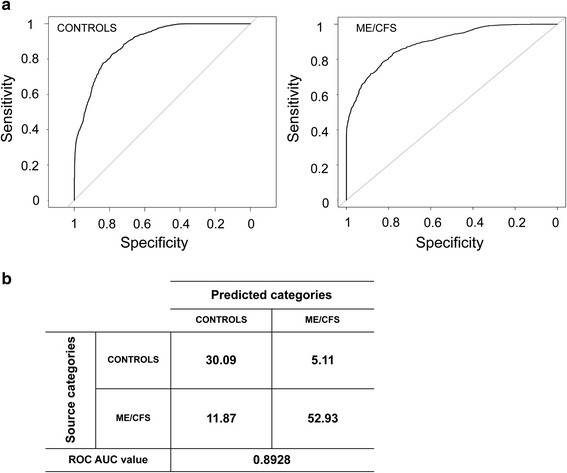
Receiver operating characteristic curves (**a**) for controls and ME/CFS patients determined using the inflammatory markers and sequencing datasets (even sampled at 32,233 sequences) and a supervised learning approach with randomForest algorithm and (**b**) confusion matrix for random forest analysis (values are presented as percentage) and ROC area under the curve (AUC) value for 97 % OTUs collapsed at the genus level. Mean AUC ROC value for five times repeated, 10-fold cross validation

Using a machine learning approach, samples were mostly successfully classified into healthy and ME/CFS groups, with the highest proportion of samples correctly classified when genus-level taxa along with data from the inflammatory markers were used in the analysis. With 97 % ID OTUs used in the analysis, 82 % of the samples could be correctly classified (standard deviation of 0.14). With OTUs collapsed at the species level, the average accuracy was 0.80 with a standard deviation of 0.11. Collapsing taxonomy to the genus level, individuals with ME/CFS were classified correctly and separately from the healthy group with an average success rate of 0.82 ± 0.12. The receiver operating curves, the AUC ROC value for the ME/CFS samples (0.89), and the confusion matrix are presented in Fig. 6. The feature importance scores for the genus-level analysis, which shows the relative importance of clinical values and microbial abundances, are available in Additional file 3: Table S1. Additionally, processing microbial sequencing data without including BMI and blood inflammatory marker levels results in 70, 75, and 72 % classification accuracy for genus, species, and OTU-level data respectively (confusion matrices available in Additional file 4: Figure S3).

## Discussion

Our analysis of the microbiome in cases suggests that gastrointestinal tract of ME/CFS patients is a pro-inflammatory environment. This environment might cause damage to the intestinal epithelium, thus augmenting microbial translocation (MT) and subsequently triggering an immune response. It has been previously documented that disruption of mucosal barrier function occurs in ME/CFS as demonstrated by the increased serum concentrations of IgA and IgM to LPS of Gram-negative enterobacteria [19]. Our data supports the hypothesis of increased MT in the ME/CFS group as evidenced by (i) significantly raised levels of plasma LPS and (ii) significantly higher levels of sCD14 and LBP, as indicators of direct LPS stimulation. Increased gut permeability and increased LPS levels have been also described in patients with liver diseases, alcoholic, and nonalcoholic steatohepatitis [23], during chronic HIV infection [24], and in inflammatory bowel disease (IBD) [25, 26] suggesting that an activation of pro-inflammatory and endotoxin-signaling cascades could be important for disease progression in ME/CFS. Consequently, high plasma LPS levels in ME/CFS could result from an increased production of endotoxin upon changes in the gut microbiota. Furthermore, we observed that sCD14 levels positively correlated with levels of LPS, LBP, and hsCRP. If there is damage to the gut mucosa, microbial translocation could increase, altering antimicrobial regulators and dysregulating the innate immune system.

As a marker, LPS is limited to particular microbes, as it is only present in Gram-negative bacteria. sCD14 is produced primarily by macrophages and hepatocytes in response to LPS but is also stimulated by other bacterial and viral agents [27]. LBP functions as a co-factor along with sCD14 and is constitutively synthesized in hepatocytes to recognize LPS released to the bloodstream but various inflammatory factors such as IL22, IL-6, and TNF-α can induce its expression [28, 29]. Nevertheless, we found significantly more patients with elevated levels of these biomarkers in comparison to the healthy group, suggesting that more MT occurs in people affected by ME/CFS.

Using both aerobic and anaerobic culturing methods, Butt and colleagues were the first to present evidence of altered fecal microbiota in ME/CFS patients compared to healthy individuals [30]. Subsequently, using culture methods and metabolite analysis, Sheedy et al. [11] obtained information concerning the fecal microbiome in patient and health cohorts. Both found that D-lactic acid-producing *Enterococcus* and *Streptococcus* species were strongly over-represented in ME/CFS patients and that among anaerobic bacteria, *Prevotella* was a bacterial genus found to be in excess in subjects with ME/CFS.

Recently, a study used high-throughput 16S rRNA gene sequencing to investigate the presence of specific alterations in the gut microbiota of ME/CFS patients from Belgium and Norway [12]. The authors amplified the V5 and V6 hypervariable 16S rRNA regions and sequenced the amplicons using a Roche FLX 454 sequencer, which resulted in an average of only 6000–7000 reads/sample. In contrast, we amplified the V4 hypervariable region of the 16S rRNA gene, sequenced amplicons using the MiSeq Illumina platform, obtained an average of many more reads/sample (98,000), and compared the resulting sequences to a different database, the Greengenes non-redundant reference database [31]. Our analysis showed that within-sample diversity is lower in the ME/CFS specimens compared to controls. The same indices in the Fremont et al. [12] study did not differ between ME/CFS and healthy subjects [12], likely due to the lower read number they obtained. Lower richness has also been observed in unhealthy or inflammatory states [32, 33] and has been associated with IBD, necrotizing enterocolitis [34], and greater abdominal discomfort levels in patients with food intolerances [35, 36].

Regardless of disease state, bacteria belonging to the Firmicutes, Bacteroidetes, Proteobacteria, and Actinobacteria phyla represented the vast majority of sequences identified. We observed reduced levels of members of the dominant phylum Firmicutes, also noted repeatedly in Crohn’s disease patients [13, 37, 38]*.* Proteobacteria were more abundant in ME/CFS patients than in controls, observed as well in inflammatory bowel disease (IBD) patients [39, 40]. In an inflamed gut, infiltrating macrophages and neutrophils release sulfur- and nitrogen-derived metabolites such as tetrathionate and nitrate [41–44]. Opportunistic members of the Proteobacteria can take advantage of the host inflammatory response by using these compounds as electron acceptors [43, 44] to generate energy and foster their own growth in the gut. We did not collect information concerning diet of patients and thus do not know whether this factor might have affected the composition and/or metabolism of the colonic microbiota in our cohorts.

We observed significantly lower levels of the genus *Faecalibacterium*, a member of the Ruminococcaceae in the ME/CFS population. For example, *Faecalibacterium prausnitzii*, which produces an anti-inflammatory protein [45], is reduced in ME/CFS cases relative to controls. This genus is also depleted in IBD [13, 38] and ulcerative colitis [46] and has been shown to have anti-inflammatory properties both in vitro and in vivo [37]. *Faecalibacterium* belongs to a group of producers of butyrate, a short chain fatty acid known to have anti-inflammatory properties and to protect the intestine [40]. Individuals with IBD and IBS [47] exhibit a lack of butyrate-producing bacteria and lower levels of butyrate in their gut [48, 49] which modulates different processes including hormone and cytokine secretion (e.g., leptin, IL-10) and activation of immune/inflammatory responses [50–52].

We also found a decrease in *Bifidobacterium*, previously observed in IBS [53–57], IBD [58], and type II diabetes [59]. Bifidobacteria are a group of lactic acid-producing bacteria that are widely used as probiotics and as targets for prebiosis [60]. Treatment with *Bifidobacterium infantis* 35624 was reported to reduce CRP levels in a cohort of ME/CFS patients [61].

We have employed a supervised machine learning approach to help prediction of disease state based on the microbiome sequence datasets [62]. Using this approach, we were able to classify unlabeled samples with some degree of accuracy, as demonstrated by the high AUC ROC value obtained (0.8928) at the genus level. This method has been recently used in several microbiome surveys to accurately place individuals into an IBD/healthy category [63], including ulcerative colitis (ROC AUC = 0.9225) and colonic (ROC AUC = 0.8787) or ileal Crohn’s disease (ROC AUC = 0.9699) [64]. Such an approach could therefore serve as a complement to other non-invasive diagnoses of symptoms or as an initial diagnosis to determine if the subject likely has ME/CFS. Because this is a relatively small cohort, to move to a formal diagnostic clinical application, a large cohort of ME/CFS and healthy controls would be needed to verify that classification would retain its accuracy with independent sample handling and sequencing.

## Conclusions

Taken together, our results suggest an ongoing damage to the gut mucosa, leading to increased microbial translocation in ME/CFS, which in turn could alter antimicrobial regulators and disregulate the innate immune system. Differences between the gut microbiomes of healthy individuals and patients with ME/CFS were identified in terms of relative abundance of specific genera. There is no single precise alteration of the gut microbiota in all ME/CFS patients we examined, but our data converges to support the concept of a less diverse and unstable community of bacteria in the disorder. It highlights the association of specific bacterial taxa with ME/CFS, and the identification of the underlying role of this altered commensal gut microbiota could lead to novel diagnostic and therapeutic strategies that would improve clinical outcome. Future studies may also reveal additional molecular markers that could be combined with gut microbiome information to enhance the sensitivity and specificity of ME/CFS diagnostic assays.

The cause of ME/CFS is unknown, but gut dysbiosis could be contributing to some of the symptoms and their severity. Developing therapeutic interventions aimed at reducing local inflammation, restoring gastrointestinal tract immunity and integrity and modifying the intestinal microbiome may ameliorate ME/CFS symptoms in a number of affected patients.

## Methods

### Human subjects and sample/data collection

All work involving human subjects was approved by the Cornell University Institutional Review Board. Fecal samples were collected at home by participants in 15-ml conical tubes containing RNAlater (Life Technologies, Grand Island, NY) and refrigerated prior to shipment. Upon arrival at Cornell University, the samples were divided into aliquots and stored at −80 °C until processing. Blood samples were drawn into EDTA and heparin tubes from an antecubital vein with subjects in the seated position. Samples were shipped by overnight courier from New York City to Cornell University (Ithaca). Upon receipt, samples were centrifuged at 4000 r.p.m. for 30 min to pellet blood cells, and plasma was stored at −80 °C until further analyses. BMI, age, and gender of subjects were recorded. ME/CFS subjects completed the Short Form 36 Health Survey (SF-36.org) and Bell’s Disability Scale [65]. Potential controls from the same geographic area as cases were screened by the physician for suitability as healthy controls. As indicated in Table 1, mean and median ages of cases and controls were within 5 years and the female to male ratios were similar.

### Plasma level determination of hsCRP, sCD14, LBP, LPS, and I-FABP

High-sensitive C-reactive protein (hsCRP) was measured from unhemolyzed EDTA plasma using a Chemiluminescence immunoassay on an Immulite 2000 (Siemens Medical Solutions Diagnostics, Deerfield, IL). Markers for microbial translocation, sCD14 and LBP, were measured in plasma samples by commercially available enzyme-linked immunosorbent assays (ELISA). Plasma sCD14 was quantified using the Quantikine Human sCD14 Immunoassay (R&D Systems, Minneapolis, MN), and plasma LBP was measured by LBP soluble ELISA kit (Hycult Biotechnology, Uden, The Netherlands) according to the manufacturers’ protocols. Plasma bacterial endotoxin, i.e., LPS, was measured from heparinized blood samples (Brandtzaeg) using the Limulus Amebocyte Lysate (LAL) assay (Lonza Group Ltd, Allendale, NJ). The method uses a chromogenic endpoint assay yielding data as endotoxin units (EU/ml). Briefly, 100 μl of each plasma sample was diluted in 200 μl of β-G-Blocker (Lonza Group Ltd, Allendale, NJ) to eliminate the possibility of false positives. Samples were further diluted with 100 μl of pyrogen-free water to give a final dilution of 1:4. All dilutions were prepared in pyrogen-free tubes. Samples were then placed in a water bath at 85 °C for 15 min to inactivate inhibitory plasma proteins. Results of LPS measured were expressed in picograms per milliliter (1 EU/ml = 100 pg/ml). Levels of intestinal fatty acid binding protein (I-FABP), a marker associated with enterocyte damage, were assayed using an ELISA (Hycult Biotechnology, Uden, the Netherlands) according to the manufacturer’s instructions. All the samples were run in duplicate.

### Statistical analysis of plasma markers levels

We initially performed a Shapiro-Wilk test to check if the data was normally distributed [66]. In case of violation of normality, data was log transformed and checked again for normality. Both parametric independent samples *t* test and a non-parametric Wilcoxon-Mann-Whitney *U* test were used to determine the significance of differences in each subject group. Values of *P* < 0.05 were considered statistically significant. All data from the biomarkers levels determination were processed and analyzed in SPSS Statistics Version 21 (Armonk, NY: IBM Corp).

### DNA extraction, 16S rRNA gene sequencing

Metagenomic DNA was isolated from an aliquot of **~**100 mg from each fecal sample using the PowerSoil-htp DNA isolation kit (MoBio Laboratories Ltd, Carlsbad, CA), which involves both chemical and physical lysis of the cells. We amplified 16S rRNA genes (V4 hypervariable region) from bulk DNA using the 515F and 806R primers as previously described [67] prior to sequencing. Duplicate PCR reactions of samples and extraction blanks consisted of 2.5X HotMasterMix (5-Prime, Inc., Gaithersburg, MD), 10–100 ng DNA template, and 0.05 μM of each primer. DNA amplification of samples and extraction blanks was performed on a 96-well plate with a minicycler PTC 200 (MJ Research) starting with 3-min denaturation at 94 °C, followed by 25 cycles consisting of denaturation (45 s at 94 °C), annealing (60 s at 50 °C), extension (90 s at 72 °C), and a final extension at 72 °C for 10 min. Samples were randomly distributed on the plate with no grouping for sample type. The replicate PCR reactions were combined and purified using a magnetic bead system (Mag-Bind EZPure, Omega Bio-Tek, Norcross, GA). PCR amplicons were quantified using the QuantiT PicoGreen dsDNA Assay Kit (Invitrogen, Carlsbad, CA). Aliquots of amplicons (at equal masses) were combined for a final concentration of approximately 15 ng/μl. Extraction blanks showed no amplification. All amplicons were then sequenced on a single run using the Illumina MiSeq 2x250 bp platform at Cornell Biotechnology Resource Center Genomics Facility.

Quality filtering and analysis of the 16S rRNA gene sequence data were performed with QIIME 1.9.0 as previously described [68]. Briefly, matching paired-end raw sequences (mate-pairs) were merged using the fastq-join command in the ea-utils software package (http://code.google.com/p/ea-utils), and merged sequences with less than a 200-bp overlap were filtered out of the dataset. The remaining merged sequences were quality filtered and assigned to samples based on their barcodes using the default parameters of QIIME. Sequences were assigned to 97 % ID OTUs by comparing them to a non-redundant reference database of near-full length sequences [31]. All OTUs that were observed fewer than two times, i.e., singletons, were removed from the analysis. The OTU table was rarefied to the sequence count of the sample with the lowest sequence depth, 32,223 sequences per sample, and used in all subsequent analyses. For statistical comparisons of healthy individuals to those afflicted with ME/CFS, *p* values obtained with the Wilcoxon-Mann-Whitney *U* test were corrected for multiple comparisons using the false discovery rate of Benjamini and Hochberg, implemented in the QIIME pipeline. We used both the weighted and unweighted UniFrac distance metrics as measures of between-sample (beta) diversity and applied principal coordinates analysis (PCoA) to visualize patterns of diversity. Within-samples (alpha) diversity was calculated using three different measures (1) ChaoI index [69]; (2) Shannon Index [70]; and (3) Phylogenetic Diversity [71].

### LEfSe analysis and machine learning

Linear discriminant effect size analysis (LEfSe) on filtered datasets [72] was performed at the genus level to find features (genera) differentially represented between healthy and ME/CFS groups. LEfSe combines the standard tests for statistical significance (Kruskal-Wallis test and pairwise Wilcoxon test) with linear discriminate analysis. It ranks features by effect size, which put features that explain most of the biological difference at top. LEfSe analysis was performed under the following conditions: the α value for the factorial Kruskal-Wallis test among classes was 0.05 and the threshold on the logarithmic LDA score for discriminative features was 2.0.

A machine learning approach was used to identify variables discriminating the two groups of samples (feature selection). For these analyses, we used either 97 % OTUs, or taxa abundances based on combining OTUs at the species and genus levels. Classification of samples as healthy controls or ME/CFS was carried out by using a random forest approach with supervised learning [73] and area under the curve (AUC) calculation to optimize feature (e.g., abundance of a particular genus) selection, implemented in the software package R. Scripts, required packages, and instructions for processing data are available on https://gist.github.com/walterst/2222618976a66b3fc8dd. In addition to the taxonomic abundance data, levels of inflammatory markers (BMI, sCD14, LBP, LPS, and I-FABP) were included in the analysis. Average accuracies were calculated with five repeats of 10-fold cross validation, which is intended to predict the accuracy of the model, and indicate over-fitting if significantly different than the full dataset results, by subsampling the data and testing this training subsample against the remaining data reference set.

## Abbreviations

hsCRP, high sensitivity C-reactive protein; IBD, inflammatory bowel disease; IBS, irritable bowel syndrome; I-FABP, intestinal fatty acid binding protein; LBP, lipopolysaccharide-binding protein; LPS, lipopolysaccharides; ME/CFS, myalgic encephalomyelitis/chronic fatigue syndrome; MT, microbial translocation; sCD14, soluble CD14

## Additional files

Additional file 1: Figure S1. 36-Item Short Form Health Survey (SF-36) profiles from studies reporting SF-36 scores for individuals with a ME/CFS diagnosis. (PDF 2514 kb) Additional file 2: Figure S2. Principal Coordinate Analysis (PCoA) plot of healthy controls versus subjects with ME/CFS. Distances were calculated with weighted UniFrac (a) and unweighted UniFrac (b). Data were evenly sampled at 32223 sequences per sample. (PDF 2631 kb) Additional file 3: Table S1. Feature Importance Scores for genus-level supervised learning. The feature importance score is the percentage increase in error rate when the given feature is permuted while other values remain constant. As there are only two categories, the increased error rate is equal for both categories. (XLSX 42 kb) Additional file 4: Figure S3. Confusion matrices for random forest analysis of microbial sequencing data (values are presented as %) and ROC area under the curve (AUC) values at the genus (a), species (b) and OTU (c) level. (PDF 1170 kb) Additional file 5: Table S2. Per-sample metadata mapping file used throughout the QIIME pipeline. (XLSX 61 kb)
