# Supplementary material for: Reduced diversity and altered composition of the gut microbiome in individuals with myalgic encephalomyelitis/chronic fatigue syndrome
Source: Microbiome. 2016 Jun 23;4:30. doi: 10.1186/s40168-016-0171-4 (PMC4918027; doi:10.1186/s40168-016-0171-4)
Supplement: Additional file 1: Figure S1. — 36-Item Short Form Health Survey (SF-36) profiles from studies reporting SF-36 scores for individuals with a ME/CFS diagnosis. (PDF 2514 kb) [file 40168_2016_171_MOESM1_ESM.pdf]

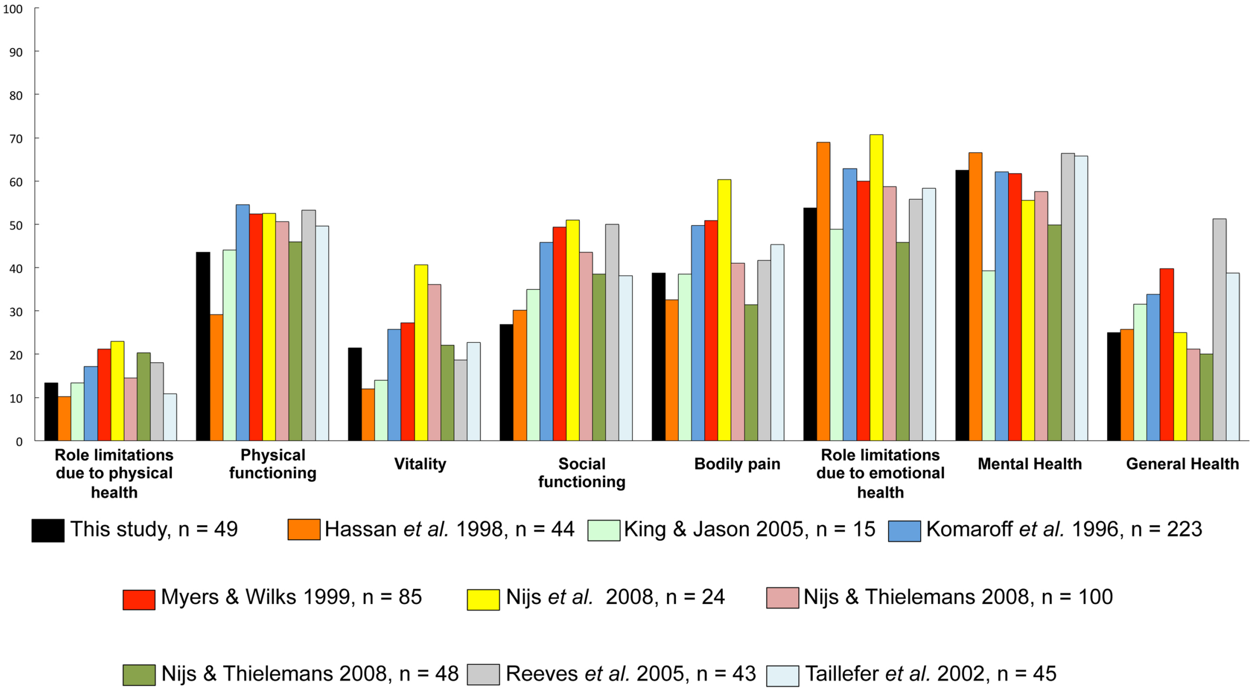
100
90
80
70
60
50
40
30
20
10
0
Role limitations due to physical health
Physical functioning
Vitality
Social functioning
Bodily pain
Role limitations due to emotional health
Mental Health
General Health
This study, n = 49
Hassan et al. 1998, n = 44
King & Jason 2005, n = 15
Komaroff et al. 1996, n = 223
Myers & Wilks 1999, n = 85
Nijs et al. 2008, n = 24
Nijs & Thielemans 2008, n = 100
Nijs & Thielemans 2008, n = 48
Reeves et al. 2005, n = 43
Taillefer et al. 2002, n = 45
