# Supplementary figures and images for: Reduced diversity and altered composition of the gut microbiome in individuals with myalgic encephalomyelitis/chronic fatigue syndrome
Source: Microbiome. 2016 Jun 23;4:30. doi: 10.1186/s40168-016-0171-4 (PMC4918027; doi:10.1186/s40168-016-0171-4)

a

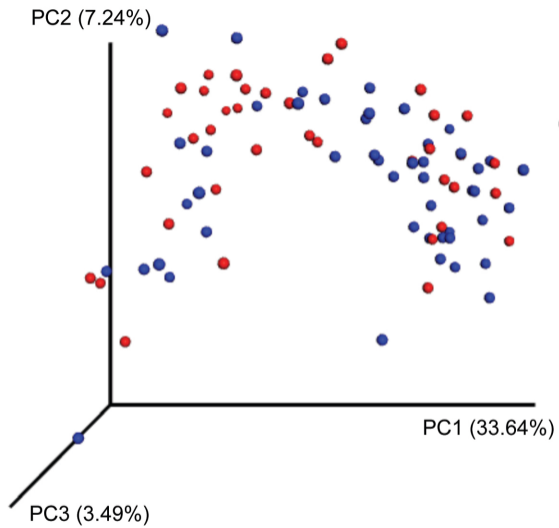

b

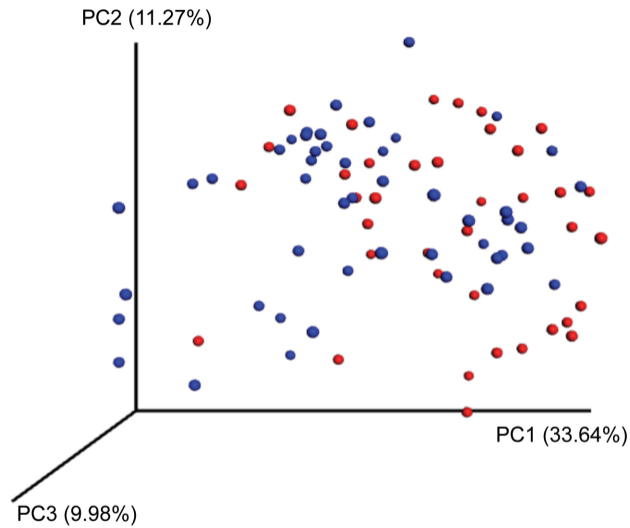

ME/CFS CONTROLS

Supplement: Additional file 2: Figure S2. — Principal Coordinate Analysis (PCoA) plot of healthy controls versus subjects with ME/CFS. Distances were calculated with weighted UniFrac (a) and unweighted UniFrac (b). Data were evenly sampled at 32223 sequences per sample. (PDF 2631 kb) [file 40168_2016_171_MOESM2_ESM.pdf]
