# Supplementary material for: Reduced diversity and altered composition of the gut microbiome in individuals with myalgic encephalomyelitis/chronic fatigue syndrome
Source: Microbiome. 2016 Jun 23;4:30. doi: 10.1186/s40168-016-0171-4 (PMC4918027; doi:10.1186/s40168-016-0171-4)
Supplement: Additional file 4: Figure S3. — Confusion matrices for random forest analysis of microbial sequencing data (values are presented as %) and ROC area under the curve (AUC) values at the genus (a), species (b) and OTU (c) level. (PDF 1170 kb) [file 40168_2016_171_MOESM4_ESM.pdf]

a.

		Predicted categories	
		CONTROLS	ME/CFS
Source categories	CONTROLS	24.86	11.63
	ME/CFS	18.60	44.90
ROC AUC value		0.7545	

b.

		Predicted categories	
		CONTROLS	ME/CFS
Source categories	CONTROLS	29.06	10.67
	ME/CFS	14.40	45.87
ROC AUC value		0.7612	

c.

		Predicted categories	
		CONTROLS	ME/CFS
Source categories	CONTROLS	24.36	8.84
	ME/CFS	19.10	47.70
ROC AUC value		0.7402	
